# *Enterococcus faecalis* bacteremia, cardiac implantable electronic device, extraction, and the risk of recurrence

**DOI:** 10.1007/s15010-022-01838-3

**Published:** 2022-05-10

**Authors:** Andreas Berge, Ludvig Arkel, Bo Nilson, Magnus Rasmussen

**Affiliations:** 1 Unit of Infectious Diseases, Department of Medicine, Karolinska Institutet, Solna, 171 76 Stockholm, Sweden; 2 Department of Infectious Diseases, Karolinska University Hospital, 171 76 Stockholm, Sweden; 3 Department of Clinical Sciences Lund, Division of Infection Medicine, Lund University, 221 00 Lund, Sweden; 4 Department of Laboratory Medicine Lund, Division of Medical Microbiology, Office for Medical Services, Lund University, Region Skåne, 221 85 Lund, Sweden; 5 Department of Clinical Microbiology, Infection Control and Prevention, Office for Medical Services, Region Skåne, 221 85 Lund, Sweden; 6 Division for Infectious Diseases, Skåne University Hospital, 221 00 Lund, Sweden

**Keywords:** *Enterococcus faecalis*, Bacteremia, Endocarditis, CIED extraction, Recurrent infection

## Abstract

**Purpose:**

In all patients with cardiac implantable electronic devices (CIED) and *Enterococcus faecalis* bacteremia (EfsB), endocarditis (IE) and CIED infection should be suspected. Guidelines recommend extraction of the CIED when CIED infection or IE is diagnosed. Whether extraction of the CIED should be done in other situations with EfsB is not known. We aimed to describe the management and outcome of patients with CIED and monomicrobial EfsB, in relation to extraction and recurrent EfsB.

**Methods:**

A population-based cohort of patients with monomicrobial EfsB from January 2014 to November 2020 was identified through microbiology registers in the Region Skåne, Sweden. Data on CIED and other clinical features were collected from medical records.

**Results:**

Among 1087 episodes of EfsB, 72 patients with CIED and monomicrobial EfsB were identified. Five of these patients were diagnosed with IE (7%), three of whom had echocardiographic changes on the CIED. Four CIED were extracted (6%). Recurrences were found in seven of 68 patients (10%) not subjected to extraction and in none of the extracted. In the group of patients without extraction, community acquisition and predisposition for IE were significantly associated with recurrent infection in univariate analyses. No infections involving the CIED were diagnosed during the recurrences.

**Conclusions:**

In patient with monomicrobial EfsB, it seems safe to omit extraction if no structural changes are found on the CIED.

**Supplementary Information:**

The online version contains supplementary material available at 10.1007/s15010-022-01838-3.

## Introduction

Gram positive bacteria are the dominating cause of infections in connection to cardiac implantable electrical devices (CIED) [1]. *Staphylococcus aureus* and coagulase negative staphylococci are the most common constituting 70 to 85% of the infections [2, 3]. *Enterococcus faecalis* is connected to approximately 4% of the CIED infections [2, 4]. *E. faecalis*, however, is a common cause of bacteremia and an important pathogen in endocarditis (IE) [5]. Risk factors for IE in *E. faecalis* bacteremia (EfsB) include monomicrobial bacteremia [6–8], but also high age, male sex, a long duration of symptom, signs of embolization, high number of positive blood cultures, an unknown origin of infection, heart valve disease, including prosthetic valve, presence of heart murmurs, a short time to blood culture positivity, and persistent bacteremia [6–13]. However, CIED is not an independent risk factor for IE in EfsB [8, 11].

International guidelines recommend extraction of the CIED in CIED infection and CIED IE [3, 14, 15]. The recommendation to extract is strong when CIED pocket infection or CIED IE with visible changes on the lead are seen on echocardiography. Most studies are made on patients with *S. aureus* bacteremia [16] and little is known on how EfsB in patients with CIED should be managed.

Our objective, in this population-based retrospective study of patients with monomicrobial EfsB and CIED, was to describe the clinical characteristics of the cohort, the management and outcome, and risk factors for recurrent infection in relation to extraction. We further aimed to be able to suggest strategies for the management of these patients.

## Material and methods

### The cohort

Information on all consecutive blood cultures positive for *E. faecalis* from January 2014 to November 2018, were obtained from the laboratory databases of Clinical Microbiology, Region Skåne, Lund, Sweden, the only laboratory in the region with a catchment area of 1.3 million inhabitants. All medical records of patients with monomicrobial EfsB older than 18 years were studied retrospectively and from patients with a CIED, detailed information was collected and stored after ethical approval obtained from the Swedish Ethics Committee (2020–00,314). Data were collected by LA and were validated by AB and MR. Some of the episodes of the present cohort have been previously described in Berge et al*.* or Oldberg et al*.* [11, 13].

### Definitions

The definition of IE and CIED infection were from Blomström-Lundqvist et al. [3], a contemporary adaptation to patients with CIED, based on the modified Duke criteria [17]. The minor criterium predisposition to IE is use according to Dajani et al*.* [18]. All changes seen on TTE or TEE, indicating IE, was considered to be caused by infection due to the difficulty to differentiate from changes due to other causes [19]. All infections fulfilling the criteria for definite IE were referred to as CIED IE irrespective whether changes were found on the CIED or heart valves [3, 17].

An episode of monomicrobial EfsB was defined by the start of the clinical symptoms and signs in a patient resulting in blood culture taken showing growth of only *E. faecalis*, however, growth of coagulase negative staphylococci in one bottle was accepted. An episode was delimited by at least 7 days of effective treatment and clinical improvement. A later clinical condition resulting in a positive blood culture with growth of *E. faecalis* within the study period of 365 days is referred to as a “recurrent infection” or “recurrence” and was not included in the study as an episode. The expression “recurrent infection” or “recurrence” is used in this study since we cannot tell whether the infection was caused by the same bacterium, indicating relapse, or by another *E. faecalis* clone indicating a reinfection.

The primary endpoint was recurrent infection with EfsB during the observation period. Origin of infection and other focal infections caused by *E. faecalis* were defined as described [11]. Comorbidities were retrieved from registrations in the medical records prior to the episode and classified according to the Charlson index modified by Quan et al*.* [20, 21]. The NOVA and DENOVA scores were calculated as described [7, 8, 11].

### Data collection and analysis

The collection of the microbiological and clinical data of an episode was from 365 days before its start until 365 days after the first positive blood culture during that episode and the extracted parameters are listed in Supplementary material. The number of CIED carriers in the Region was taken from the Swedish Pacemaker and Implantable Cardioverter-Defibrillator Registry.

The analysis of the collected data was conducted in Stata, version 15.1 (StataCorp, College Station, TX, USA). The odds ratios (OR) and their confidence intervals were calculated when applicable. To describe the differences in dichotomous variables, the *p *value of Fisher’s exact test was used. Differences between continuous variables were analyzed with Wilcoxon’s rank-sum test. Values are presented as proportions or medians with interquartile ranges (IQR).

## Results

### Description of the study cohort

**Fig. 1:**
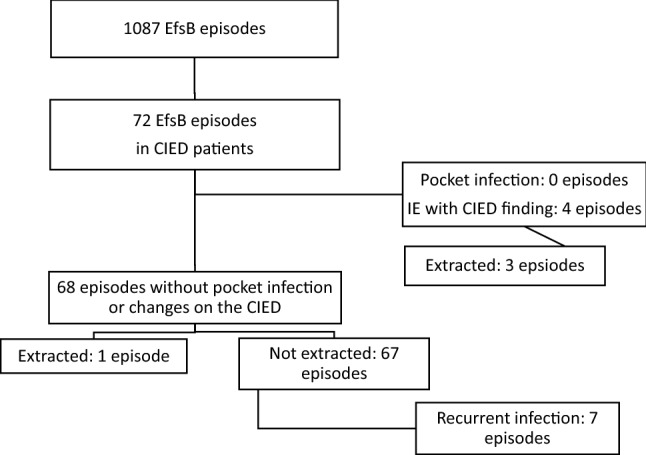
Flow chart of episodes of EfsB in patients with CIED

In the Region Skåne, 1087 episodes of EfsB were retrieved from January 2014 to November 2020. Of these, 654 were found to be monomicrobial. The criteria for inclusion as an episode in the study were fulfilled in 72 of the monomicrobial EfsB episodes. There were 9044 patients with CIED (average in 2014–2020 in the population) giving an approximate incidence of 1.1 monomicrobial EfsB episodes/1000 CIED/year (Fig. 1). The first column of Table 1 summarizes the characteristics of the cohort. In five episodes the patients were diagnosed with CIED IE, two with findings only on the CIED, two with findings on the CIED and the left side, and one with findings only on the left side of the heart. No patients were diagnosed with definite IE without having any structural findings. No patient with monomicrobial EfsB was diagnosed with a generator pocket infection (Table 1).

**Table 1:** Characteristics of the cohort of patients with CIED and EfsB and comparison of patients subjected or not subjected to extraction

Characteristics	All (*n* = 72)^a^	Extracted (*n* = 4)	Not extracted (*n* = 68)
Age (years)	80 (66–94)	84	79 (65–93)
Sex (female)	15 (21%)	1 (25%)	14 (21%)
Present CIED not the first	6 (8%)	1 (25%)	5 (7%)
CIED implantation (months)	60 (14–116)	52	60 (14–116)
Type of CIED			
PPM	57 (79%)	3 (75%)	54 (79%)
ICD	5 (7%)	0 (0%)	5 (8%)
CRT-P	5 (7%)	1 (25%)	4 (6%)
CRT-D	5 (7%)	0 (0%)	5 (8%)
Predisposition for IE^b^	16 (22%)	0 (0%)	16 (24%)
Charlson score	4 (2–5)	2 (1–4)	4 (2–5)
Acquisition			
Community	20 (28%)	1 (25%)	19 (28%)
Health care associated	36 (50%)	2 (50%)	33 (48%)
Nosocomial	16 (22%)	1 (25%)	16 (24%)
Origin of infection known	29 (40%)	1 (25%)	28 (41%)
Pocket infection	0 (0%)	0 (0%)	0 (0%)
Time to positive BC (hours)	11 (7–15)	13	11 (7–15)
NOVA-score ≥ 4	43 (60%)	3 (75%)	40 (59%)
DENOVA-score ≥ 3	21 (29%)	1 (25%)	20 (29%)
Management			
TTE performed	37 (51%)	4 (100%)	33 (49%)
TEE performed	19 (26%)	3 (75%)	16 (24%)
Positive for CIED vegetation	4 (6%)	3 (75%)	1 (1%)
IE, possible	19 (28%)	0 (0%)	19 (28%)
IE, definite	5 (7%)	3 (75%)	2 (3%)
Treatment, total, (days)	15 (11–22)	45	14 (10–18)
Outcome			
Recurrence in EfsB	7 (10%)	0 (0%)	7 (10%)
Death within 30 days	10 (14%)	0 (0%)	10 (15%)
Death within one year	29 (43%)	1 (25%)	28 (44%)

*CRT-P and CRT-D* cardiac resynchronization therapy pacemaker and defibrillator

^a^Values are given as proportions and for continuous variables as medians and IQR (not the group subjected to extraction of the CIED, due to the low number of patients)

^b^Refers to the minor criterion, predisposition, in Li et al*.* [17]

### CIED extraction

In the cohort of 72 episodes, four patients had the CIED extracted, three of whom were diagnosed with CIED IE. One patient with EfsB due to urinary tract infection (UTI) had the CIED extracted, without any IE diagnosis. The patient had a recent CIED implantation, 14 days prior to the EfsB, negative TEE, no signs of pocket infection, slow response to treatment without an explanation found, and a negative culture from the explanted CIED. The clinical characteristics of the four patients are shown, Supplementary table 1. All four had a new CIED implanted and were treated 37–62 days. None of these patients was given longtime suppressive antibiotic treatment. Clinical characteristics of those that were subjected to extraction of the CIED and those not subjected to extraction is shown in Table 1. In the 68 patients not subjected to extraction, two patients were diagnosed with IE, one of them had changes on the CIED. In both patients, the decision was taken not to extract the CIED based on the risks connected to the intervention. The patients were given 26 and 38 days of treatment in total and did not have a recurrent infection. One patient survived the study period and the other died during treatment for the IE, due to progressive cardiac failure caused by mitral valve regurgitation, massive embolization, and was not eligible for extraction of the CIED or cardiac surgery. Thirty-three (49%) of the patients were investigated with transthoracic echocardiography (TTE) and 16 (24%) with a transesophageal echocardiography (TEE) (Table 1). None of the patients who had their CIED extracted had a recurrent infection whereas seven recurrences were noted in 68 patients not subjected to extraction (the difference was not statistically significant, Table 1).

### Characteristics of patients with recurrent infection

Clinical variables in patients, not subject to extraction, who had recurrent or did not have recurrent monomicrobial EfsB were compared. Univariate analysis identified community acquisition and predisposing condition to be significantly associated to recurrent monomicrobial EfsB (Table 2). Significantly more patients were subjected to TEE in the group that got a recurrent infection. No difference in the treatment time was seen between the groups (Table 2). None of the 68 patients was given longtime antibiotic suppressive therapy. Five patients were given more than 42 days of treatment and the longest duration of therapy was 104 days.

**Table 2:** Clinical characteristics of the episodes in patients affected by a recurrent EfsB infection compared to episodes in patients without a recurrent infection

Characteristics	Later recurrence (n = 7)	No recurrence (n = 61)	Odds ratio (95% CI)	*p* value^c^
Age (years)	78 (72–90)	80 (74–87)	n/a	0.88
Sex (female)	2 (29%)	12 (20%)	1.6 (0.3–9)	0.63
Charlson score	3 (2–4)	4 (2–5)	n/a	0.35
Acquisition				**0.03**
Community acquired	5 (71%)	14 (23%)	8.4 (1.5–48)	**0.02**
Health care associated	2 (29%)	31 (51%)	0.39 (0.07–2.2)	0.43
Nosocomial	0 (0%)	16 (26%)	n/a	0.19
Present CIED not the first	0 (0%)	5 (8%)	n/a	1.0
CIED implantation (months)	71 (28–133)	57 (14–112)	n/a	0.59
Type of CIED				1.0
PPM	7 (100%)	47 (77%)	n/a	
ICD	0 (0%)	5 (8%)	n/a	
CRT-P	0 (0%)	4 (7%)	n/a	
CRP-D	0 (0%)	5 (8%)	n/a	
Predisposition for IE^a^	4 (57%)	12 (20%)	5.4 (1.07–28)	**0.048**
Prosthetic valve	3 (43%)	9 (15%)	4.3 (0.8–23)	0.1
Duration of symptoms (days)^b^	3 (1–5)	1 (1–3)	n/a	0.23
Heart murmur	3 (43%)	21 (34%)	1.4 (0.3–7)	0.69
Fever ≥ 38 degrees	5 (71%)	39 (64%)	1.4 (0.25–7.9)	1.0
Embolization	0 (0%)	3 (5%)	n/a	1.0
Origin of infection	3 (43%)	25 (41%)	1.1 (0.2–5)	1.0
Pocket infection	0 (0%)	0 (0%)	n/a	n/a
Positive DENOVA-score	4 (57%)	16 (26%)	3.8 (0.8–19)	0.18
Time to positive BC (hours)	9 (8–13)	11 (9–14)	n/a	0.23
Management				
TTE performed	2 (29%)	31 (51%)	0.39 (0.07–2.2)	0.43
TEE performed	4 (57%)	12 (20%)	5.4 (1.1–28)	**0.048**
PET-CT performed	0 (0%)	1 (1%)	n/a	n/a
IE, possible	4 (57%)	15 (26%)	4.1 (0.82–20)	0.09
IE, definite	0 (0%)	2 (3%)	n/a	1.0
Treatment, iv, (days)	4 (2–12)	2 (1–5)	n/a	0.09
Treatment, total, (days)	14 (10–16)	16 (11–18)	n/a	0.72
Outcome				
Death within 30 days	0 (0%)	10 (16%)	n/a	0.58
Death within 365 days	1 (14%)	27 (44%)	0.2 (0.02–1.9)	0.23

^a^Refers to the minor criterion, predisposition, in Li et al*.*[17]

^b^Refers to the days of symptoms preceding the medical situation resulting in taking a blood culture showing monomicrobial EfsB, D in DENOVA [11]. Values are given as proportions and for continuous variables as medians and IQR

^c^Significant differences (*p *values and *α* < 0.05) are shown in bold

One patient (14%) with a recurrent infection died within the study period and in the group without a recurrence 27 patients did not survive the study period (44%), however, this difference was not significant. None of the patients that died during the study period had indications of a recurrent infection with *E. faecalis*.

Of the seven patients with recurrent infections, four had two episodes and three patients had three or more episodes. The characteristics of patients with recurrent infections are shown (Table 3). The recurrent infection episodes were diagnosed at a median of 55 days (range 18–130 days) after the end of therapy for the preceding EfsB episode.

**Table 3:** Description of the seven recurrent infection episodes

Patient	Age	Gender	Prosthetic valve	Focus in first episode	TEE done	Time between first and second episode	Focus in second episode	TEE done	Treatment as IE	Third episode	Extraction	Deceased during study
1	92	Male	No	Unknown	No	144	Unknown	No	No	SD	No	No
2	71	Male	No	Unknown	Yes	34	Unknown	Yes	No	No	No	No
3	83	Female	Yes	Unknown	Yes	147	Unknown	Yes	No	Yes	Yes	No
4	72	Female	Yes	Unknown	Yes	49	IE and AGI	Yes	Yes	No	No	No
5	76	Male	No	UTI	No	53	UTI	No	No	No	No	No
6	90	Male	No	UTI	Yes	66	Unknown	No	No	Yes	No	No
7	78	Male	Yes	UTI	No	99	Unknown	Yes	No	No	No	Yes

*SD* spondylodiscitis, *AGI* aortic graft infection

Three patients were diagnosed with UTI during the first episode. In one of the patients, a urinary tract malignancy was diagnosed during the recurrent infection and the patient died due to that on day 299.

In four patients, no origin of infection was diagnosed during the first episode. During the following episodes, one patient was diagnosed with spondylodiscitis (patient 1). Patient 4 was diagnosed with a CIED IE, during the second episode, utilizing PET-CT to indicate involvement of an aortic prosthetic valve and the aortic graft but not of the CIED. That patient was neither subjected to CIED extraction nor thoracic surgery due to the infection and was treated for CIED IE followed by lifelong suppressive antibiotic treatment. She survived during the study period. Patient 3, had a prosthetic valve and suffered three episodes of bacteremia without known focus. During the third episode, the CIED was extracted but with negative culture. The patient was given lifelong oral suppressive therapy and survived the study period.

## Discussion

In this retrospective population-based study of patients with CIED and affected by monomicrobial EfsB, we identify 72 episodes. In four patients (6%) the CIED was extracted and no recurrent infections were seen in these patients. Further, we found recurrent infection with EfsB in seven of 68 (10%) of the episodes where the CIED was not extracted. In none of the recurrences, changes were seen on the CIED, so we found no proof that the recurrent infections were related to the CIED. One patient got the CIED extracted during the recurrence, without microbiologic data verifying an infection. The main conclusion is that data from this study supports that extraction of the CIED could be omitted if no changes are seen on the CIED when examined with TEE or other modalities.

CIED infection or IE must be suspected in patients with CIED, presenting with bacteremia with bacterial species prone to cause IE. For instance, in patient with *S. aureus* bacteremia, the guidelines suggest extraction of the CIED also without signs of pocket infection or CIED IE due to the risk as such [3, 15, 22]. Only small groups of CIED carriers with EfsB have been described previously [8, 11] why the outcome in terms of CIED infection, CIED IE or pocket infection, extraction of the CIED, and recurrent infections is not well known. To our knowledge, this is the largest study on the subject and the population-based approach of this study indicates that it, in a Swedish context at least, is representative for the clinical practice. The all-cause mortality in the cohort is high, probably due to old age and comorbidity, but mortality attributable to the CIED infection or CIED IE was not found. The clinical practice described in the study follows to a high extent the recommendations to extract the CIED when CIED changes are seen and the criteria for CIED IE are fulfilled but far from all of the patients were examined with TEE (24%). The short treatment times, the lack of use of oral longtime suppressive antibiotic therapy, and that the percentage of recurrent infections is not higher than other studies speaks against that CIED infections were missed in our cohort.

We have previously suggested the DENOVA score to be used in monomicrobial EfsB to decide whether TEE should be done. However, in this study, the DENOVA score does not have the sensitivity to identify all, but 3 out of 5, patients with CIED IE. The two patients not identified by DENOVA had growth in all blood cultures and an unknown origin of infection but were devoid of other risk factors. The DENOVA score does not include CIED because it was not an independent risk factor in the multivariate analysis. Further, CIED is neither included in the predisposition for IE in the DENOVA-score nor as a minor criteria in the diagnostic criteria [3, 17, 18, 22]. However, CIED is a risk factor for IE in bacteremia with other species, for instance *S. aureus* [13, 23, 24], why this would be interesting to further study in *E. faecalis*.

Although this is the largest study cohort of CIED-carrying patients with EfsB, it has obvious limitations. Due to the limited size of the group of patients subjected to extraction, the study does not have the power to show if extraction of the CIED is superior to non-extraction. The retrospective design and far from complete evaluation using TEE, make it possible that some patients with changes on the CIED could have been missed. Moreover, only two patients were subjected to PET-CT, also possibly contributing to under-diagnosis. Furthermore, some patients could have died of an undiagnosed IE, another undiagnosed EfsB infection, or a recurrent infection. Finally, we do not know if the recurrent infections are true relapses or reinfection with another clone.

Despite the shortcomings, we believe that the observation of low frequency of CIED infections in monomicrobial EfsB has implications for the management of such patients[25], [26],[27].

## Supplementary Information

Below is the link to the electronic supplementary material.

Supplementary file1 (DOCX 29 kb)

## References

[1] Sohail MR, Uslan DZ, Khan AH (2007). Management and outcome of permanent pacemaker and implantable cardioverter-defibrillator infections. J Am Coll Cardiol.

[2] Hussein AA, Baghdy Y, Wazni OM (2016). Microbiology of cardiac implantable electronic device infections. JACC Clin Electrophysiol.

[3] Blomström-Lundqvist C, Traykov V, Erba PA (2020). European heart rhythm association (EHRA) international consensus document on how to prevent, diagnose, and treat cardiac implantable electronic device infections-endorsed by the heart rhythm society (HRS), the Asia Pacific heart rhythm society (APHRS), the Latin American heart rhythm society (LAHRS), international society for cardiovascular infectious diseases (ISCVID) and the European society of clinical microbiology and infectious diseases (ESCMID) in collaboration with the European association for cardio-thoracic surgery (EACTS). Eur J Cardiothorac Surg.

[4] Oh TS, Le K, Baddour LM (2019). Cardiovascular implantable electronic device infections due to enterococcal species: clinical features, management, and outcomes. Pacing Clin Electrophysiol.

[5] Olmos C, Vilacosta I, Fernández-Pérez C (2017). The evolving nature of infective endocarditis in Spain: a population-based study (2003–2014). J Am Coll Cardiol.

[6] Fernández-Guerrero ML, Herrero L, Bellver M, Gadea I, Roblas RF, de Górgolas M (2002). Nosocomial enterococcal endocarditis: a serious hazard for hospitalized patients with enterococcal bacteraemia. J Intern Med.

[7] Bouza E, Kestler M, Beca T (2015). The NOVA score: a proposal to reduce the need for transesophageal echocardiography in patients with enterococcal bacteremia. Clin Infect Dis.

[8] Dahl A, Lauridsen TK, Arpi M (2016). Risk factors of endocarditis in patients with enterococcus faecalis bacteremia: external validation of the NOVA score. Clin Infect Dis.

[9] Maki DG, Agger WA (1988). Enterococcal bacteremia: clinical features, the risk of endocarditis, and management. Medicine (Baltimore).

[10] Dahl A, Iversen K, Tonder N (2019). Prevalence of infective endocarditis in enterococcus faecalis bacteremia. J Am Coll Cardiol.

[11] Berge A, Krantz A, Ostlund H, Naucler P, Rasmussen M (2019). The DENOVA score efficiently identifies patients with monomicrobial enterococcus faecalis bacteremia where echocardiography is not necessary. Infection.

[12] Oldberg K, Rasmussen M (2021). Enterococcus faecalis in blood cultures-a prospective study on the role of persistent bacteremia. Diagn Microbiol Infect Dis.

[13] Oldberg K, Thorén R, Nilson B, Gilje P, Inghammar M, Rasmussen M (2021). Short time to blood culture positivity in enterococcus faecalis infective endocarditis. Eur J Clin Microbiol Infect Dis.

[14] Baddour LM, Wilson WR, Bayer AS (2015). Infective endocarditis in adults: diagnosis, antimicrobial therapy, and management of complications: a scientific statement for healthcare professionals from the American heart association. Circulation.

[15] Sandoe JA, Barlow G, Chambers JB (2015). Guidelines for the diagnosis, prevention and management of implantable cardiac electronic device infection. Report of a joint working party project on behalf of the British society for antimicrobial chemotherapy (BSAC, host organization), British heart rhythm society (BHRS), British cardiovascular society (BCS), British heart valve society (BHVS) and British society for echocardiography (BSE). J Antimicrob Chemother.

[16] Sohail MR, Palraj BR, Khalid S (2015). Predicting risk of endovascular device infection in patients with *Staphylococcus aureus* bacteremia (PREDICT-SAB). Circ Arrhythm Electrophysiol.

[17] Li JS, Sexton DJ, Mick N (2000). Proposed modifications to the Duke criteria for the diagnosis of infective endocarditis. Clin Infect Dis.

[18] Dajani AS, Bisno AL, Chung KJ (1990). Prevention of bacterial endocarditis. Recommendations by the American heart association. JAMA.

[19] George MP, Esquer Garrigos Z, Vijayvargiya P (2020). Discriminative ability and reliability of transesophageal echocardiography in characterizing cases of cardiac device-lead vegetations versus non-infectious echodensities. Clin Infect Dis.

[20] Charlson ME, Pompei P, Ales KL, MacKenzie CR (1987). A new method of classifying prognostic comorbidity in longitudinal studies: development and validation. J Chronic Dis.

[21] Quan H, Li B, Couris CM (2011). Updating and validating the Charlson comorbidity index and score for risk adjustment in hospital discharge abstracts using data from 6 countries. Am J Epidemiol.

[22] Habib G, Lancellotti P, Antunes MJ (2015). ESC guidelines for the management of infective endocarditis: the task force for the management of infective endocarditis of the European society of cardiology (ESC). Endorsed by: European association for cardio-thoracic surgery (EACTS), the European association of nuclear medicine (EANM). Eur Heart J.

[23] Palraj BR, Baddour LM, Hess EP (2015). Predicting risk of endocarditis using a clinical tool (PREDICT): scoring system to guide use of echocardiography in the management of *Staphylococcus aureus* bacteremia. Clin Infect Dis.

[24] Tubiana S, Duval X, Alla F (2016). The VIRSTA score, a prediction score to estimate risk of infective endocarditis and determine priority for echocardiography in patients with *Staphylococcus aureus* bacteremia. J Infect.

[25] Friedman ND, Kaye KS, Stout JE (2002). Health care–associated bloodstream infections in adults: a reason to change the accepted definition of community-acquired infections. Ann Intern Med.

[26] Bizzini A, Durussel C, Bille J, Greub G, Prod'hom G (2010). Performance of matrix-assisted laser desorption ionization-time of flight mass spectrometry for identification of bacterial strains routinely isolated in a clinical microbiology laboratory. J Clin Microbiol.

[27] Sonesson A, Öqvist B, Hagstam P, Björkman-Burtscher IM, Miörner H, Petersson AC (2004). An immunosuppressed patient with systemic vasculitis suffering from cerebral abscesses due to *Nocardia farcinica* identified by 16S rRNA gene universal PCR. Nephrol Dial Transplant.

